# Substrate Specificity Diversity of Human Terminal Deoxynucleotidyltransferase May Be a Naturally Programmed Feature Facilitating Its Biological Function

**DOI:** 10.3390/ijms25020879

**Published:** 2024-01-10

**Authors:** Aleksandra A. Kuznetsova, Svetlana I. Senchurova, Anastasia A. Gavrilova, Timofey E. Tyugashev, Elena S. Mikushina, Nikita A. Kuznetsov

**Affiliations:** 1 Institute of Chemical Biology and Fundamental Medicine, Siberian Branch of Russian Academy of Sciences (SB RAS), 8 Prospekt Akad. Lavrentyeva, Novosibirsk 630090, Russia; sandra-k@niboch.nsc.ru (A.A.K.); svetasenchurova@gmail.com (S.I.S.); a.gavrilova@alumni.nsu.ru (A.A.G.); tyugashev@niboch.nsc.ru (T.E.T.);; 2 Department of Natural Sciences, Novosibirsk State University, 2 Pirogova Str., Novosibirsk 630090, Russia

**Keywords:** human terminal deoxynucleotidyltransferase, V(D)J recombination, substrate specificity, dNTP recognition, metal ion, cofactor, enzyme kinetics

## Abstract

Terminal 2′-deoxynucleotidyl transferase (TdT) is a unique enzyme capable of catalysing template-independent elongation of DNA 3′ ends during V(D)J recombination. The mechanism controlling the enzyme’s substrate specificity, which is necessary for its biological function, remains unknown. Accordingly, in this work, kinetic and mutational analyses of human TdT were performed and allowed to determine quantitative characteristics of individual stages of the enzyme–substrate interaction, which overall may ensure the enzyme’s operation either in the distributive or processive mode of primer extension. It was found that conformational dynamics of TdT play an important role in the formation of the catalytic complex. Meanwhile, the nature of the nitrogenous base significantly affected both the dNTP-binding and catalytic-reaction efficiency. The results indicated that neutralisation of the charge and an increase in the internal volume of the active site caused a substantial increase in the activity of the enzyme and induced a transition to the processive mode in the presence of Mg^2+^ ions. Surrogate metal ions Co^2+^ or Mn^2+^ also may regulate the switching of the enzymatic process to the processive mode. Thus, the totality of individual factors affecting the activity of TdT ensures effective execution of its biological function.

## 1. Introduction

DNA polymerases play a key part in the maintenance of genome stability by participating in such processes as repair, replication and recombination [1,2]. During the functioning of any DNA polymerase, a series of molecular events occur sequentially, including DNA and dNTP binding, which induce mutual conformational changes in both the DNA backbone and the protein globule [3,4,5]. The formation of a catalytically active complex is accompanied by the attachment of a nucleotide to the free 3′-OH end of the template, subsequent translocation of the enzyme along the DNA molecule and a release of the pyrophosphate residue [6,7]. It should be noted that overall efficiency of DNA synthesis substantially depends on the processes that occur after the addition of a nucleotide to the 3′-OH end of the template. At this stage, the DNA polymerase can move along the DNA by one nucleotide to perform the next cycle of nucleotide addition in the case of processive DNA synthesis, or the enzyme can dissociate from the extended DNA substrate, thereby switching to the mode of distributive DNA synthesis.

Conformational dynamics of the enzyme–substrate complex make an important contribution to high accuracy of DNA polymerases [8] by ensuring the selectivity of the enzyme towards the incoming dNTP; this selectivity is based both on initial recognition of the structure of the incoming nucleotide and on the process of the formation of a transition state, accompanied by considerable conformational changes in the structure of the enzyme. In this context, the incoming nucleotide enters into electrostatic interactions with charged amino acid residues, thus facilitating the transition of the enzyme–substrate complex from the open conformation to a closed one. The contacts arising during these processes depend on the correct geometry of the resulting base pairs, which is based on the assembly of a network of hydrogen bonds, on the stacking of complementary base pairs and on the formation of contacts between the incoming nucleotide and a template base.

Terminal 2′-deoxynucleotidyl transferase (TdT), which belongs to the class of DNA polymerases, is a unique enzyme capable of conducting template-independent DNA synthesis [9,10]. Accordingly, in the mechanism of action of this enzyme, there are no restrictions related to the initiation of complementary interactions. This ability of the enzyme is required for V(D)J recombination, during which TdT adds 1 to 10 random nucleotides to the free 3′-OH end of the V segment, resulting in higher immunological heterogeneity [11]. Furthermore, TdT can attach structurally diverse derivatives of nucleotides [12,13], implying an ability to correctly bind modified nucleotides in the dNTP-binding site. Nonetheless, even if natural nucleotides are incorporated into the growing strand, TdT can catalyse this process with different efficiency and processivity [14,15].

Despite intensive research on structural features of the TdT enzyme [16], it is currently unknown how the active site of the enzyme can recognise nucleoside triphosphates that differ significantly in their type and structure and can use them as substrates in the polymerisation reaction. In this regard, we have previously examined the kinetics of DNA elongation under the action of TdT as well as performed molecular modelling of the processes taking place in the ternary catalytic enzyme–substrate complex TdT–DNA_n_–dNTP and in the post-catalytic TdT–DNA_n+1_ complex [15]. The results showed that DNA synthesis processivity—which is characterised by the efficiency of enzyme translocation along the DNA strand after the addition of a new nucleotide and is regulated by the ratio of translocation and dissociation rate constants—significantly depends on the nature of the nitrogenous base in the incoming nucleotide. Additionally, the rate constant of the catalytic reaction depends significantly on the nature of the cofactor metal ion. For instance, in the presence of Mg^2+^ ions, the incorporation efficiency decreased in the series dGTP > dTTP ≈ dATP > dCTP in that study; in this context, in the case of dGTP, the primer was extended in the processive mode, whereas in the case of other dNTPs, the primer was extended in the distributive mode. Moreover, the incorporation of dCTP was very inefficient. By contrast, in the presence of Mn^2+^ ions, the mode of the elongation of dATP and dTTP changed from distributive to processive, which significantly increased the efficiency of their attachment. Furthermore, a comparison of the rate constants showed that Mn^2+^ ions raise the observed catalytic rate constant of dNTP attachment by ~10-fold, regardless of the nature of the nitrogenous base. The results of molecular dynamics (MD) simulations obtained in that work indicated that, upon recognition of various nucleotides, TdT comes into specific contacts with the nucleotide being incorporated. It was found that guanine forms a wide network of contacts with amino acid residues in the active-site pocket, which allow dGTP to be efficiently placed into the appropriate position and facilitate the catalytic reaction.

It should be pointed out that the kinetic mechanism of action of TdT, including rate constants of elementary steps of the enzymatic process, is still unclear, although such mechanisms have been determined for a number of template-dependent DNA polymerases [17,18,19]. Nevertheless, the absence of a template strand can certainly have a significant effect both on parameters of the DNA- and nucleotide-binding steps and on the stages of catalysis, translocation and dissociation of the product. In addition, insights into kinetic aspects of the mechanism of termini elongation and into substrate specificity of TdT during V(D)J recombination can shed light on patterns of the course of this complicated, highly organised, molecular process.

In this regard, in this work, we performed a pre-steady-state kinetic analysis of conformational transitions of human TdT and DNA during the catalytic cycle. The conformational dynamics of the interacting molecules were recorded in real time by the stopped-flow method. Conformational alterations in the enzyme in the course of interaction with various nucleoside triphosphates were registered by means of changes in fluorescence intensity of Trp residues. To study the conformational dynamics of DNA, we used a fluorescent analogue of cytosine (pyrrolocytosine, C^Py^) within the nucleoside triphosphate. It was demonstrated that the binding of dNTP and its correct positioning in the active site are key steps in the assembly of a catalytically active complex, and it is these steps that substantially affect both the efficiency of attachment of nucleotides of different structures and the processivity of this attachment. Bioinformatic analysis of nucleotidyl transferases from 469 organisms and examination of the structure of the dNTP-binding site of these enzymes allowed us to identify several possibly important amino acid residues that can influence the efficiency of binding and attachment of dNTPs. Finally, five mutants of human TdT were investigated, and it was shown that residues Asp395 and Glu456 can determine the specificity of the enzyme to the nucleotide being incorporated, substantially affect the enzymatic activity in the presence of Mg^2+^ ions and thus—along with the effect of surrogate Co^2+^ or Mn^2+^ ions—regulate the transition of the enzymatic process to the processive mode.

## 2. Results and Discussion

### 2.1. Conformational Dynamics of TdT

Previously, it has been reported [15] that the nature of the cofactor metal ion has a strong impact on the incorporation of dNTP into the growing DNA primer strand under the action of TdT. In this context, in the presence of Mn^2+^ ions, considerable acceleration of the enzymatic process was documented in comparison with the ‘natural’ ion cofactor Mg^2+^. Therefore, in the present work, Mn^2+^ ions were chosen to analyse the pre-steady-state kinetics of the polymerase reaction catalysed by wild type (WT) TdT. Using the stopped-flow method, the enzyme and substrate samples were mixed for ~1 ms, and then changes in fluorescence intensity of tryptophan residues of TdT were recorded. The changes in fluorescence intensity of tryptophan residues in the enzyme characterise conformational transitions in the protein molecule.

Analysis of structural data [16] showed that in the absence of a DNA primer, the aromatic ring of Trp450 is parallel to (and is partially in a stacking interaction with) the nitrogenous base of the incoming dNTP. In this context, in the ternary complex of the enzyme with the primer and dNTP, the nitrogenous base of the incoming nucleotide is in a stacking interaction with the nitrogenous base of the nucleotide located at the 3′ end of the DNA primer, whereas the Trp450 residue does not come into contact with dNTP. Therefore, it can be hypothesised that during the interaction of the enzyme with the DNA primer and dNTP, the environment of this Trp residue will change, thereby making it possible to register a change in fluorescence intensity of Trp during a conformational rearrangement of this enzyme region.

**Figure 1:**
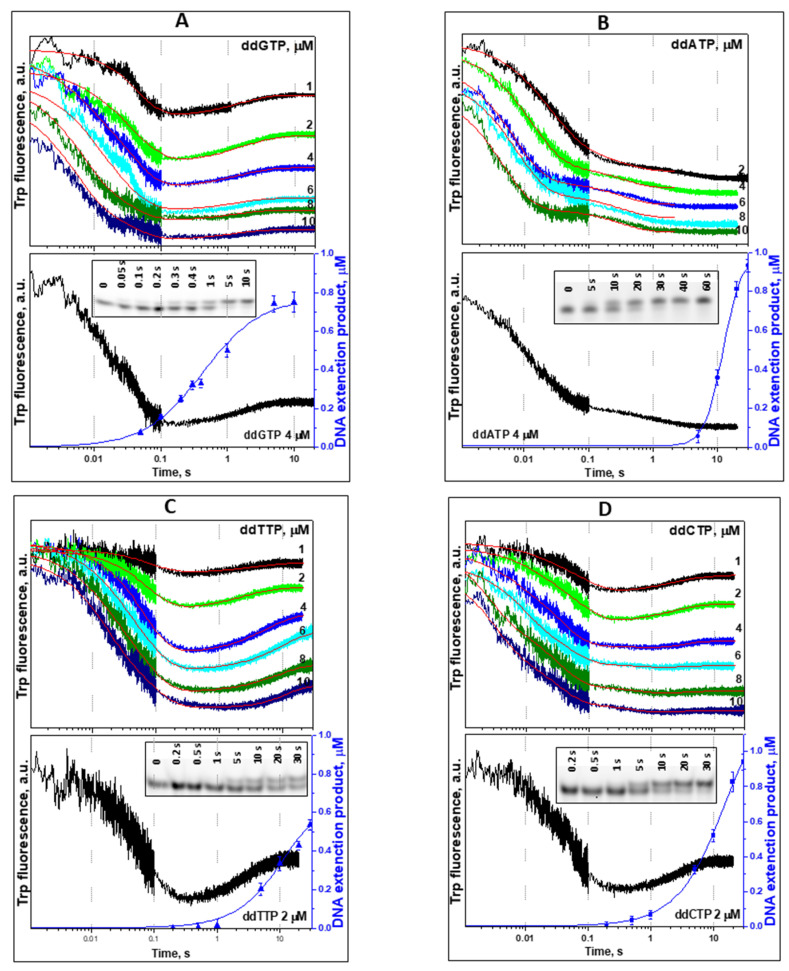
Changes in fluorescence intensity of Trp residues during the interaction of pre-formed complex [WT TdT•DNA_ss_] with ddGTP (**А**), ddATP (**B**), ddTTP (**C**) or ddCTP (**D**). DNA_ss_ is a single-stranded oligodeoxynucleotide (primer). Coloured traces represent experimental data; red curves denote theoretical fitting. The lower panels depict superposition of kinetic curves characterising a change in fluorescence intensity of Trp and the accumulation of products of ddNTP attachment. The concentration of TdT (2 µM), DNA_ss_ (1 µM), and ddNTP is indicated in the figure.

Figure 1 shows changes in fluorescence intensity of Trp residues during the binding and attachment of ddNTP. As is evident in the obtained kinetic curves, the process of binding and attachment of ddNTP to the primer is characterised by a rapid decrease and subsequent growth of fluorescence intensity of Trp residues. Furthermore, a polyacrylamide gel electrophoresis (PAGE) analysis of the kinetics of accumulation of products of ddNTP attachment revealed that the increase in fluorescence intensity of Trp residues coincides with accumulation of the polymerisation reaction product. Nonetheless, it must be mentioned that the TdT enzyme features rapid fluorescence burnout under irradiation (photobleaching). For this reason, all the obtained kinetic curves were corrected via Equation (1) to adjust the data for this effect.

Of note, with ddATP, no increase in fluorescence intensity of Trp residues was observed (Figure 1B). According to the electrophoretic analysis, however, the accumulation of the polymerisation reaction product in the presence of ddATP was observed at time points greater than 5 s. It is possible that this phenomenon is due to specific features of the interaction of adenine with residue Trp450 and an effect on its fluorescence. Additionally, it was found that TdT undergoes considerable photobleaching during the irradiation, which prevents signal registration at time points later than 10–20 s. Consequently, processes occurring after 10 s cannot be registered in this way. Apparently, the photobleaching of the enzyme was the reason why it was not possible to register the catalysis stage in the case of slowly attached ddATP (Figure 1).

**Scheme 1:**
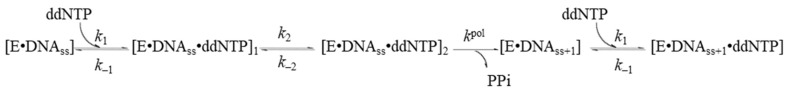
The kinetic mechanism of binding and incorporation of ddNTP into the primer under the action of WT TdT.

The resultant kinetic curves of changes in fluorescence intensity of Trp residues were described by means of the kinetic mechanism shown in Scheme 1, which includes two equilibrium stages of binding of ddNTP and one irreversible stage corresponding to the formation of the reaction product as well as the second cycle of binding of ddNTP. The rate constants for this kinetic Scheme 1 are presented in Table 1. For ddATP, the kinetic curves up to time point 2 s were described by a kinetic scheme containing two equilibrium steps of ddATP binding; in this case, rate constants were obtained only for the formation of the enzyme•primer•ddATP ternary complex. The rate of incorporation of ddATP into the DNA primer was determined via PAGE.

The kinetic curves of accumulation of the polymerase reaction product were fitted to an exponential function; the dependence of the observed rate constant of product formation on the concentration of ddNTP was hyperbolic. The dependence of the observed rate constant on the enzyme concentration was fitted to Equation (2), and the obtained values of constants ^PAAG, dATP^*K*_d_ and ^PAAG, dATP^*k*^pol^ are given in Table 1.

In Scheme 1, [E•DNA_ss_] is a pre-formed complex of WT TdT with the DNA primer, [E•DNA_ss+i_•ddNTP]_n_ represents various enzyme–substrate complexes, and [E•DNA_ss+1_] is a complex of WT TdT with an extended DNA primer containing ddN at the 3′ end.

*K*^dNTP^_d_ decreases in the order ddTTP > ddCTP ≈ ddGTP > ddATP, while *k*_pol_ decreases in the order ddGTP > ddCTP > ddTTP > ddATP. The obtained data indicated a considerable influence of the nature of the nitrogenous base both on the formation of the ternary complex and on the catalytic stage. Nevertheless, it can be said that overall efficiency of dNTP incorporation (dTTP ≈ dCTP > dATP > dGTP) under the conditions of several enzyme turnovers, as determined earlier [15], is very similar to the series of *K*^dNTP^_d_ changes.

For instance, for ddATP, readers can see that despite the efficient formation of the initial complex (*K*_1_ is the highest), *k*_2_ is 1–2 orders of magnitude lower than that for the other ddNTPs, thus leading to the smallest *K*_2_. The second equilibrium stage in Scheme 1 corresponds to a conformational adjustment of the enzyme to the dNTP being incorporated; this process is most efficient for ddCTP and ddGTP. The catalytic rate constant is the highest for ddGTP, which, along with the efficient course of the first two steps, makes dGTP the best substrate for TdT.

### 2.2. DNA Conformational Dynamics: Binding and Attachment of dC^Py^TP

**Scheme 2:**
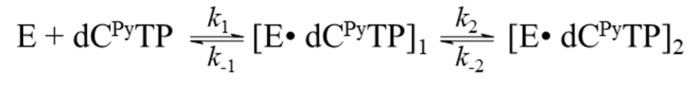
The kinetic mechanism of the binding of dC^Py^TP to TdT.

**Figure 2:**
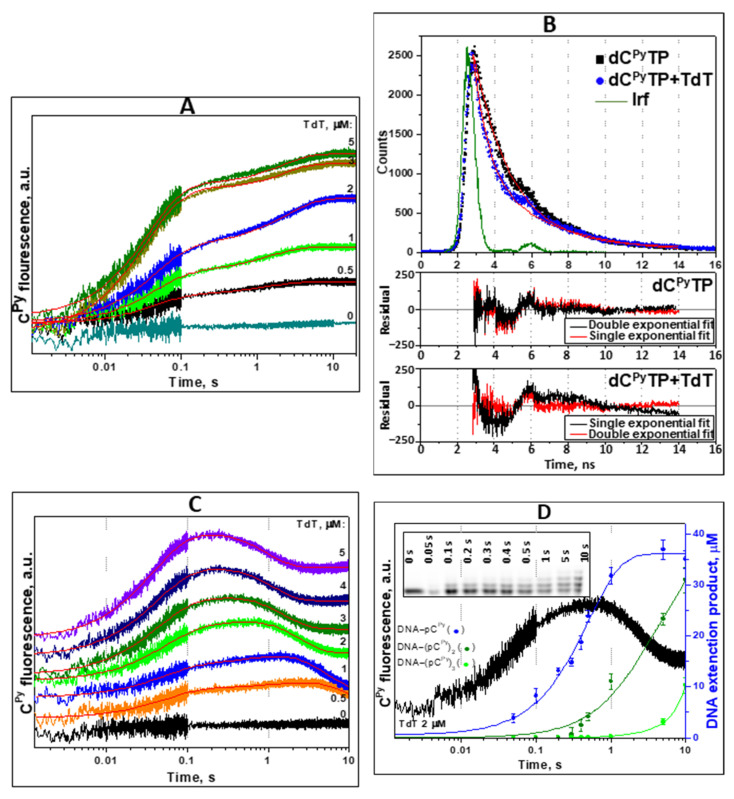
(**A**) Changes in C^Py^ fluorescence intensity during the interaction of dC^Py^TP with TdT. (**B**) The fluorescence decay curve obtained for free dC^Py^TP (black squares) and for the [dC^Py^TP•TdT] complex (blue circles). (**C**) Change in C^Py^ fluorescence intensity during the interaction of dC^Py^TP with the pre-formed [TdT•DNA_ss_] complex. [dC^Py^TP] = 3.0 µM, [DNA_ss_] = 1.0 µM, and the concentration of TdT is indicated in the figure. Coloured traces represent experimental data; red curves denote theoretical fitting. (**D**) Superposition of kinetic curves characterising the change in C^Py^ fluorescence intensity and the accumulation of polymerisation products.

To examine the conformational dynamics of DNA during the catalytic cycle, 2′-deoxyribopyrrolocytosine triphosphate (dC^Py^TP) was employed. Binding of dC^Py^TP in the absence of a DNA primer resulted in a biphasic increase in pyrrolocytosine fluorescence intensity (Figure 2A). The observed changes in fluorescence intensity of pyrrolocytosine were described by means of kinetic Scheme 2, which involves two equilibrium stages. The obtained constants are listed in Table 2. One can see that parameter *K*_1_ of the first stage is close to the *K*_1_ value determined for pyrimidine nucleotide triphosphates (Table 1). On the other hand, the second stage is characterised by high *K*_2_, which indicates the stability of the final [E•dC^Py^TP]_2_ complex. It can be theorised that the first stage corresponds to the formation of the initiation complex of the enzyme with dC^Py^TP, whereas the second slow phase of the increase in fluorescence intensity apparently reflects the assembly of a more thermodynamically favourable complex of the enzyme with a nucleotide triphosphate in the absence of a DNA primer strand.

The existence of two possible states of dC^Py^TP within the nucleotide-binding site of TdT is also supported by time-resolved flowmetry data (Figure 2B). For free dC^Py^TP, the fluorescence decay curve was described as a single-exponential equation, and fluorescence lifetime was 2.2 ns, in good agreement with the literature data [20,21]. By contrast, for dC^Py^TP bound to TdT, the fluorescence decay curve was described as a two-exponent equation, and fluorescence lifetime was 0.85 and 3.6 ns. These data indicated that in the complex with the enzyme, the fluorophore exists in two different environments. In this context, the extension of fluorescence lifetime—relative to free dC^Py^TP—is possible due to the shielding of the fluorophore molecule from the solvent within the complex with the enzyme. On the other hand, the state in which the fluorophore has substantially shorter fluorescence lifetime as compared to free dC^Py^TP is probably due to the interaction of the pyrrolocytosine residue with hydrophilic residues of the nucleotide-binding site of TdT.

In Scheme 2, E is the enzyme and [E•dC^Py^TP]_n_ represents various enzyme–substrate complexes.

**Scheme 3:**
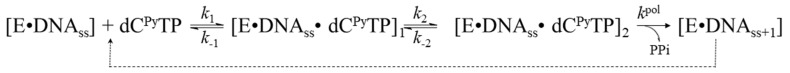
The kinetic mechanism of binding and incorporation of dC^Py^TP into the DNA primer under the influence of TdT.

In the presence of a DNA primer, the binding of dC^Py^TP should be accompanied by the incorporation of a fluorescent nucleotide in a multi-turnover mode. Indeed, the obtained kinetic curves contained not only the phase of the increase in fluorescence intensity of C^Py^, but also an additional phase of a signal decrease following it (Figure 2C). Meanwhile, at low concentrations of TdT, the growth phase had two well-pronounced stages. Therefore, the kinetic mechanism describing this process, aside from the two stages of binding of dC^Py^TP, according to Scheme 2, should include a step of attachment of a nucleotide to the primer and a transformation of the complex of the enzyme with the extended product, thereby giving rise to a new reaction complex (Scheme 3). To confirm the nature of the additional phase of the decrease in C^Py^ fluorescence intensity, the kinetics of accumulation of products of dC^Py^TP attachment were analysed via PAGE (Figure 2D). Notably, the phase of the decrease in C^Py^ fluorescence intensity coincided with the accumulation of the reaction product corresponding to the attachment of the second dC^Py^ residue to the primer. Thus, the combination of the fluorescence and PAGE data allowed us to conclude that Scheme 3 is the minimum possible kinetic mechanism that describes the entire cycle of binding and attachment of dC^Py^TP to the primer. All the obtained values of individual constants are shown in Table 2.

A comparison of the rate constants (Table 2) of the formation of the initial complex between TdT and dC^Py^TP indicated that the DNA primer almost does not affect the rate of formation of this complex but stabilises it by ~3-fold due to a decline in rate constant *k*_−1_. In this context, rate constants of the second stage of dC^Py^TP binding increase both in the forward and reverse directions, together causing a ~6-fold drop of *K*_2_. Despite the difference in the parameters of the individual stages of binding of dC^Py^TP, total dissociation constant *K*^dCPyTP^_d_ for the complex of the enzyme with dC^Py^TP has similar values when we compare the absence and presence of the DNA primer. Furthermore, the fluorescent analysis of the entire enzymatic cycle helped us to calculate catalytic rate constant *k*_pol_, which proved to be even higher than the rate constant of incorporation of the most effective nucleotide, ddGTP (Table 1).

Additionally, the rate of incorporation of dC^Py^TP into the DNA primer was determined in experiments on separation of the primer extension products via PAGE. Kinetic curves of the accumulation of the polymerase reaction product corresponding to the attachment of the first pyrrolocytosine residue to the DNA primer were approximated to an exponential equation. The dependence of the observed rate constant on the enzyme concentration had a hyperbolic shape and was fitted to Equation (2). Despite the large error from determining the parameters in this way, the obtained values of the total dissociation constant *K*^dCPyTP^_d_ and the rate constant of the catalytic stage, *k*_pol_, were consistent with the findings of the analysis of fluorescent data (Table 2).

In Scheme 3, [E•DNA_ss_] is a pre-formed complex of TdT with the DNA primer, [E•DNA_ss_•dC^Py^TP]_n_ represents ternary enzyme–substrate complexes, and [E•DNA_ss+1_] is a complex of TdT with an extended DNA primer.

The totality of our kinetic data enabled us to propose a sequence of mutual adjustments between the enzyme and the substrate during of nucleoside triphosphate binding and its attachment. For example, in the process of interaction of dNTP with the [TdT•DNA_ss_] complex, after the formation of the initial [TdT•DNA_ss_•dNTP]_1_ complex, it is ‘reorganised’ into the [TdT•DNA_ss_•dNTP]_2_ complex; the rearrangement is necessary for the correct orientation of dNTP in the active site of the enzyme (a decrease in Trp fluorescence intensity until time point 100 ms). In the meantime, the nature of the nitrogenous base has a considerable impact on both stages of the binding (Table 1). According to MD simulations [15], a nitrogenous base forms a network of contacts with amino acid residues in the active-site pocket, while the number and efficiency of these contacts depend on the nature of the nitrogenous base. Apparently, the stage-by-stage formation of these contacts takes place precisely during the two registered kinetic stages. It can be hypothesised that at the second stage, specific contacts arise between amino acid residues of the enzyme’s active site and the nitrogenous base contained in the nucleoside triphosphate, thus leading to the assembly of a thermodynamically stabler enzyme–substrate complex: [TdT•DNA_ss_•dNTP]_2_. Additionally, in ref. [15], guanine turned out to be the most coordinated nitrogenous base, and this property allows dGTP to be effectively placed in an optimal position within the active-site pocket and facilitates the catalytic stage of attachment to the primer (growth in Trp fluorescence intensity up to time point 1 s). If we examine the full cycle of binding and attachment of dC^Py^TP—which, just as dGTP, is an example of a ‘processive’ substrate (Table 2)—readers can see that the binding and attachment of subsequent nucleoside triphosphates is an efficient process, indicating the absence of rate-limiting factors for the dissociation of pyrophosphate or for translocation of the enzyme on the extended primer molecule. That is, after the catalytic stage, the next nucleoside triphosphate is rapidly bound and incorporated, and this process ensures the functioning of the enzyme in the processive mode even in the presence of Mg^2+^ ions.

Thus, our findings indicate that the binding of dNTP and its correct placement into the active site are the key steps during the formation of the catalytically active complex, and it is these steps that have a strong effect both on the efficiency of attachment of nucleotides of different structures and on the processivity of the incorporation.

### 2.3. Functional Residues in the Active Site of TdT That Ensure Substrate Specificity of the Enzyme

**Figure 3:**
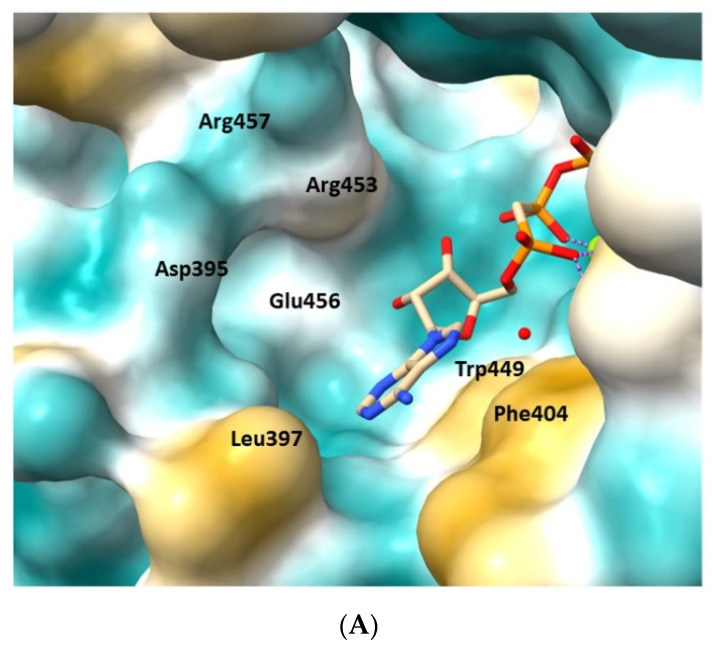

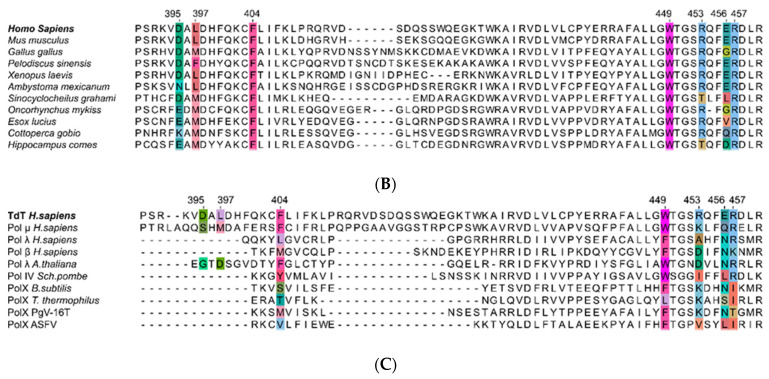
(**A**) Architecture of the dNTP-binding pocket in the complex of TdT with AMPcPP (Protein Data Bank [PDB] ID 4i2d; the amino acid residue numbering matches that of human TdT). (**B**) Alignment of TdT orthologs, which illustrates the variability of amino acid positions corresponding to TdT Asp395, Leu397, Phe404, Trp449, Arg453, Glu456 and Arg457. (**C**) Alignment of selected PolX family DNA polymerases, which illustrates the variability of amino acid positions corresponding to TdT Asp395, Leu397, Phe404, Trp449, Arg453, Glu456 and Arg457 in the whole PolX family.

To determine molecular reasons for the differences in the binding and attachment efficiency among nucleoside triphosphates, the structure of the dNTP-binding pocket was examined next. The analysis of the structural data [16,22] showed that one side of the dNTP-binding pocket is lined with hydrophobic residues Leu397, Phe404 and Trp449 (Figure 3A, the residue numbering corresponds to human TdT), while the other side of the pocket is formed by hydrophilic amino acid residues Asp395, Arg453, Glu456 and Arg457. Despite such a bipolar architecture of the dNTP-binding pocket, it has previously been revealed by MD simulations [15] that a nitrogenous base mainly interacts with hydrophilic amino acid residues, thereby creating a network of hydrogen bonds.

To identify the potential roles of all the amino acid residues that constitute the dNTP-binding pocket, we aligned 469 amino acid sequences of TdT enzymes from various organisms (Supplementary Materials). Figure 3B depicts a representative sample of these sequences that includes the most common differences at positions Asp395, Leu397, Phe404, Trp449, Arg453, Glu456 and Arg457 among TdT enzymes from all 469 species, and Table 3 shows overall frequency of amino acid residues at the respective positions. Furthermore, we aligned amino acid sequences of human TdT and of more distant X-family DNA polymerases (similar to enzymes Pol μ, Pol β and Pol λ), which show properties of template-dependent enzymes in gap-filling DNA repair processes (Figure 3C).

It was found that the positional equivalent of Asp395 is occupied by aspartate residues in 72.3% of the examined TdT sequences, with glutamate residues taking up the remaining 27.3% among the 469 analysed species (Supplementary Materials), with rare exceptions, such as a lysine residue found in *C. gobio* and an asparagine residue in both *X. laevis* and *A. mexicanum*. Of note, this residue is optional for gap-filling DNA polymerases of the PolX family (Figure 3C). Asp395 is poorly resolved in crystal structures capturing mimics of pre- or post-catalytic complexes between TdT and single-strand DNA primers; either the sidechain only is resolved or the entire residue is unresolved, with the exception of the Zn^2+^-co-soaked ternary complex structure [16]. In crystal structures of TdT complexes with double-stranded DNA, the Asp395 residue is bound to either the 5′-OH group of the 3′-terminal base of the template strand (PDB ID 5D46) or to the 3′-sub-terminal guanine base of the template strand (PDB ID 5D49) [22]. The role of Asp395 has been examined [23], and the researchers noticed slightly weaker polymerase activity in the D395A mutant together with altered substrate specificity: dGTP incorporation was slower relative to the other three nucleosides. Additionally, it has been found by MD simulations that Asp395 engages in stable hydrogen bonding with all types of nucleobases [15].

The Leu397 residue of human TdT proved to be nonconserved among the 469 enzymes, with retention of only the volume and relative hydrophobicity after replacement with a leucine, methionine or phenylalanine residue, which could be present at this position (Figure 3B). The sidechain of position 397, together with conserved Phe404, acts as a wedge disrupting the stacking interaction of the 3′-terminal base with the upstream base. In comparative assays of primer elongation activity performed on nine diverse vertebrate Leu-TdTs and five avian Met-TdTs, as well as the murine TdT L398M mutant, Lu et al. observed that the methionine residue at position 397 gives higher polymerase activity [24]. Phe404 is strictly conserved among TdTs (Figure 3B) but not in the whole PolX family (Figure 3C). It is reported that either of two substitutions L397A and F404A results in a drastic reduction in the transferase activity of TdT [25]. On the other hand, no notable difference has been observed in interactions of this mutant with nucleotide-competitive inhibitors [26].

The Trp449 residue and the preceding glycine also were found to be strictly conserved among TdT enzymes (Figure 3B) and among Pol μ enzymes (Figure 3C) and form a so-called GW motif, in contrast to the YF motif of Pol β and Pol λ [27,28]. GW/YF motifs serve as a deterrence to ribonucleotide incorporation via a steric clash between the backbone chain of the Tyr residue and the 2′-OH group of the incoming rNTP [29,30,31]. In TdT and Pol μ, the respective glycine residue of the GW motif cannot discriminate between ribonucleotide and 2′-deoxyribonucleotide incorporation [32,33]. Indeed, the ability of both mutants Pol β Y271A and Pol λ Y505A to discriminate against ribonucleotide insertion is an order of magnitude weaker [30,34], whereas the Pol μ G433Y mutant manifests drastically stronger sugar discrimination [29].

Glu456 is conserved among 76.3% of the examined TdT sequences, with glycine taking up ~20% of instances. Of note, in the PolX family, an amino acid residue at this position interacts with the base of the incoming dNTP [35], but this position has substantially higher diversity among members of the whole PolX family (Figure 3C).

Both Arg453 and Arg457 also proved to be highly conserved. On the other hand, an MD simulation [15] indicates that the guanidine group of Arg453 enters into hydrogen bonds with H-donor groups of nitrogenous bases G, A and T; by contrast, residue Arg457 forms a network of contacts only with pyrimidine nitrogenous bases. Notably, an increase in summarised relative lifetime of the H-bond between pyrimidine nucleotides and residues Arg453 and Arg457 was observed [15] in the post-catalytic complex in comparison with the pre-catalytic ternary complex, thereby supporting stabilisation of the enzyme–product complex and potentially lowering the enzyme’s efficiency owing to product inhibition.

Thus, our analysis of amino acid sequences and literature data on likely properties of the amino acid residues that form the dNTP-binding pocket suggests that these residues have high functional significance in processes of molecular recognition of individual nitrogenous bases, and the combination of these residues underlies the differences in specificity of the enzyme when various nucleotides are compared.

### 2.4. Alterations (by Site-Directed Mutagenesis) of Substrate Specificity of TdT towards the Inserted dNTP

An in-depth mutational analysis of two amino acid residues located in the dNTP-binding pocket, namely Asp395 and Glu456, was performed to experimentally test their influence on the specificity of the enzyme towards the nucleotide being incorporated. For this purpose, mutants of TdT were generated containing either a single substitution (D395N, D395E or E456N) or a double substitution (D395N/E456N or D395K/E456Q). These amino acid substitutions were chosen for relocating a negative charge (variant D395E), neutralising it (variant D395N), neutralising the charge and expanding the internal space (variants E456N and D395N/E456N) or even creating a positively charged region (variant D395K/E456Q) in the dNTP-binding pocket.

**Figure 4:**
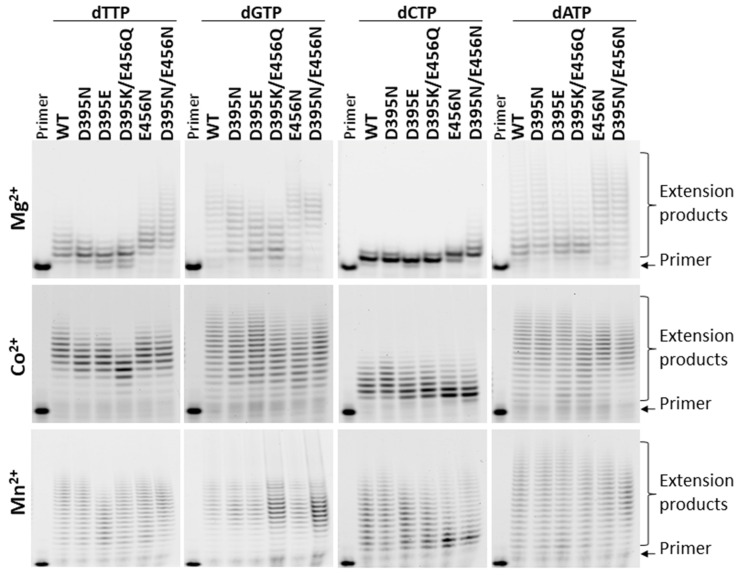
A comparison of substrate specificity between WT TdT and its mutants D395N, D395E, E456N, D395K/E456Q and D395N/E456N in the presence of Mg^2+^, Co^2+^ or Mn^2+^. [TdT] = [DNA] = 1.0 μM, [dNTP] = 10 μM, and reaction duration was 1 min.

The resultant mutants were used to evaluate the efficiency of dNTP incorporation as compared to the WT enzyme. Furthermore, a comparison of substrate specificity between WT TdT and the mutants was performed at a 10-fold excess of dNTP in the presence of various cofactor ions, namely Mg^2+^, Co^2+^ or Mn^2+^ (Figure 4).

Analysis of PAGE data suggested that in the presence of Mg^2+^ ions, the charge neutralisation by the D395N substitution causes slight deterioration in incorporation processivity of dTTP, dGTP and dATP, but not dCTP, in which case the enzyme functions in the distributive mode. Relocation of the negative charge by D395E substitution or its replacement by a positive charge (via D395K/E456Q double substitution) led to an even more pronounced loss of incorporation processivity for the three dNTPs but also had little effect on distributive synthesis in the case of dCTP. It is worth mentioning that the effect of a single substitution at position 395 became negligible when the Co^2+^ ion served as a cofactor, which, in comparison with Mg^2+^, improved overall efficiency of processive synthesis in the case of dTTP, dGTP and dATP, whereas for dCTP, it even switched the synthesis from the distributive to processive mode. Nevertheless, the positive charge seen after the D395K/E456Q double substitution, even in the presence of Co^2+^, still slightly diminished efficiency as compared to single substitutions at position 395. Moreover, with the Mn^2+^ cofactor ion, which enhanced the processive synthesis even more appreciably, the differences between the WT enzyme and its mutants became modest.

Another set of mutants, namely E456N and D395N/E456N, overall showed expansion of the internal space and charge neutralisation inside the dNTP-binding pocket. Each of these substitutions improved the processivity of attachment of dN as compared to the WT even in the presence of Mg^2+^ ions. For example, with these mutants (E456N or D395N/E456N), there were primer extension products representing the attachment of up to 10 dT residues and up to six dC residues to the primer, whereas the WT enzyme attached no more than six and three such residues, respectively. In the presence of surrogate ions (Co^2+^ or Mn^2+^), efficient incorporation of 8–10 or more residues was observed in the processive mode, regardless of the nature of the nucleoside triphosphate for both mutants of the enzyme (E456N and D395N/E456N). On the one hand, these data confirm our previous finding [15] that cofactor Me^2+^ can considerably change parameters of primer elongation by strongly affecting the rate of nucleotide attachment and the polymerisation mode. On the other hand, the use of various cofactor ions helped to visualise the impact of each amino acid substitution on incorporation efficiency of dNTPs.

**Figure 5:**
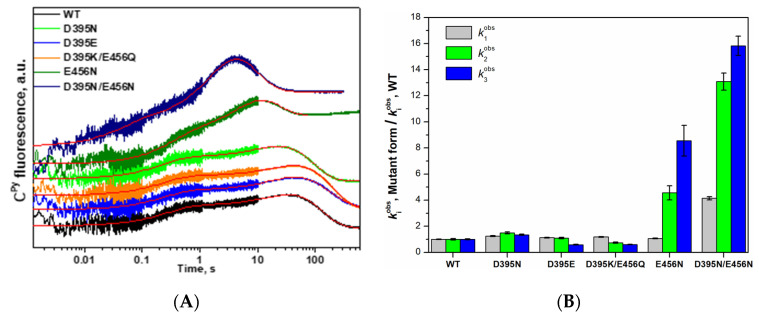
(**A**) Changes in C^Py^ fluorescence intensity in the course of the interaction of dC^Py^TP with the pre-formed [E•DNA_ss_] complex in the presence of Mg^2+^ ions. [dC^Py^TP] = 3.0 µM, [DNA_ss_] = [TdT] = 1.0 µM. Coloured traces represent experimental data; red curves denote theoretical fitting. (**B**) Relative activity of mutants as determined by normalisation of the observed rate constants.

To determine the steps of the enzyme–substrate interaction that are affected by substitutions of amino acid residues in the dNTP-binding pocket, an assay of the attachment of dC^Py^TP to a DNA primer was performed in the presence of Mg^2+^ ions, in which case the greatest differences in the processivity were seen among the mutants (Figure 5A). For all mutants, just as for the WT enzyme, two successive phases of fluorescence intensity growth were detectable, characterising the stages ‘binding of dC^Py^TP’ and ‘formation of a catalytic complex’, as well as a phase of signal decline coinciding with the attachment of the second fluorescent nucleotide to the primer. The resulting kinetic curves were fitted to a sum of three exponentials, which made it possible to calculate observed rate constants for each phase of the change in C^Py^ fluorescence intensity. The relative activity of the mutants of the enzyme was determined through normalisation of an observed rate constant of a mutant to the corresponding rate constant from the WT enzyme (Figure 5B).

A comparison of the relative efficiency of each stage of the interaction showed that the neutralisation of the charge at position 395 by D395N substitution, relocation of the charge (variant D395E) or switching the charge to a positive one (variant D395K/E456Q) only insignificantly affects the efficiency of both the step of binding of dC^Py^TP and its attachment. At the same time, with D395E or D395K/E456Q, there was a slight decrease (less than two-fold) in the observed rate constant of nucleotide attachment to the primer, in agreement with the results of PAGE (Figure 4).

On the other hand, charge neutralisation and expansion of the internal space of the pocket by E456N substitution did not affect the initial binding of dC^Py^TP but improved the efficiency of the formation of the catalytic complex and the efficiency of the catalytic stage by ~4-fold and ~8-fold, respectively. The synergistic effect of charge neutralisation at positions 395 and 456, in combination with the expansion of the internal volume (variant D395N/E456N), substantially increased the efficiency of all three stages of the process: the binding of dC^Py^TP by ~4-fold, the formation of the catalytic complex by ~13-fold and the addition reaction by ~16-fold. Such a strong increase in efficiency at each stage seems to be key to the switching of the enzyme from the distributive to processive mode.

## 3. Materials and Methods

### 3.1. Protein Purification and Mutagenesis

WT TdT (the gene was inserted into plasmid pET28c) was purified as reported elsewhere [15]. The single and double mutants of TdT were produced by site-directed mutagenesis using a set of primers (Table S1) and Pfu DNA polymerase (SibEnzyme, Novosibirsk, Russia). All mutated genes were sequenced to ensure the presence of the desired mutation. All mutants were purified in the same manner as for the WT TdT enzyme. SDS-PAGE with Coomassie Blue staining revealed a purity of >95%.

### 3.2. Nucleoside Triphosphates and DNA_ss_ Primer

Unlabelled Ultrapure Grade dNTPs were purchased from SibEnzyme (Novosibirsk, Russia), and dNTP from GlenResearch (Headquarters, Sterling, VA, USA). 5′-FAM (6-carboxyfluorescein)-labelled or unlabelled oligonucleotides 5′-FAM-GGAAGA-3′/5′-GGAAGA-3′ were used as the DNA_ss_ primer [15].

### 3.3. Stopped-Flow Fluorescence Experiments

Single-turnover experiments were conducted to examine the transient kinetics associated with incorporation of a single nucleotide (in the case of ddNTP) or of dC^Py^TP into a DNA_ss_ primer. Stopped-flow fluorescence measurements were carried out on a model SX.18MV stopped-flow spectrometer (Applied Photophysics, Leatherhead, Surrey, UK) as described before [36,37]. General principles of investigation into conformational dynamics in real time using the stopped-flow method were recently described in detail [38]. Each kinetic curve was averaged over at least five independent experiments. The instrument’s dead time is 1.38 ms. To detect intrinsic Trp fluorescence, λ_ex_ 290 nm and λ_em_ > 320 nm (filter WG 320, Schott, Mainz, Germany) were chosen. To detect C^Py^ fluorescence, λ_ex_ 340 nm and λ_em_ > 370 nm (filter LG-370-F, Corion, USA) were used. All stopped-flow experiments were conducted with an unlabelled DNA_ss_ primer at 37 °C in reaction buffer composed of 50 mM Tris-HCl pH 8.0 and 1 mM MnCl_2_. When Trp fluorescence was measured, the enzyme concentration was 2 µM, the DNA_ss_ primer concentration was 1 µM, and ddNTP concentrations were varied from 1 to 10 µM. When C^Py^ fluorescence was studied, the DNA_ss_ primer concentration was 1 µM, dC^Py^TP concentration was 3 µM, and the enzyme concentration was varied from 0.5 to 5 µM. The TdT mutants’ activity assay for dC^Py^TP incorporation was performed in the presence of 5 mM MgCl_2_ or 1 mM MnCl_2_. In this case, the enzymes’ concentration was 1 µM. The irradiation of the enzyme with UV light leads to disturbances in tryptophan residues accompanied by a decrease in fluorescence intensity (photobleaching). To adjust the measured data for the photobleaching effect, fluorescence intensities were processed using Equation (1).

(1) $$F=\left(\left(F_{\mathrm{obs}}-F_{0}\right)\times e^{k_{\mathrm{bleach}}\times t}\right)+F_{0}$$

where F is the adjusted fluorescence intensity, F_obs_ is observed fluorescence intensity, F_0_ is background fluorescence and *k*_bleach_ is a coefficient determined for each substrate concentration.

### 3.4. Quenched-Flow Experiments

Pre-steady-state (rapid quench) experiments were carried out in a KinTek Quench-Flow machine (KinTek Corp., Snow Shoe, PA, USA). All quenched-flow experiments were conducted with a FAM-labelled DNA_ss_ primer at 37 °C in reaction buffer consisting of 50 mM Tris-HCl pH 8.0 and 1 mM MnCl_2_ or 5 mM MgCl_2_ or 1 mM CoCl_2_. It should be noted that Mn^2+^ and Co^2+^ are widely recognized cofactors for TdT and are used in a number of works together with Mg^2+^ [23,39]. In [15], concentrations of ions [Mg^2+^] = 5 mM, [Mn^2+^] = 1 mM and [Co^2+^] = 1 mM were found to be optimal for the reaction catalysed by hTdT. Products were analysed via electrophoresis in a denaturing 15% polyacrylamide gel and visualised by means of the fluorescence of the FAM fluorophore using an E-Box CX.5 TS gel documentation system (Vilber Lourman). A kinetic analysis of the primer elongation by TdT in the presence of MnCl_2_ was conducted via the following procedure. Reaction mixtures (70 µL) contained 50 mM Tris-HCl (pH 8.0), 1 mM MnCl_2_, 1 µM DNA_ss_ primer, 2–10 µM dNTPs and 2 µM WT TdT. The reaction was initiated by the addition of the enzyme and was allowed to proceed at 37 °C; aliquots (10 µL) were withdrawn as required and mixed with 10 µL of a stop solution (9 M urea and 25 mM EDTA).

The TdT mutants’ activity assay for dNTP incorporation was performed in the presence of 1 mM MnCl_2_ or 5 mM MgCl_2_ or 1 mM CoCl_2_ using 1 µM enzyme, 1 µM DNA_ss_ primer and 10 µM dNTPs. The TdT mutants’ activity assay for dC^Py^TP incorporation was performed in the presence of 1 mM MnCl_2_ or 5 mM MgCl_2_ using 1 µM enzyme, 1 µM DNA_ss_ primer and 3 µM dC^Py^TP. After incubation at 37 °C for 10 s or 1 min, the primer extension reaction was stopped by the addition of the stop solution (9 M urea and 25 mM EDTA).

### 3.5. C^Py^ Fluorescence Lifetime Measurement

Fluorescence decay curves for free dC^Py^TP and for the [dC^Py^TP•TdT] complex in 50 mM Tris-HCl buffer pH 8.0 were obtained at 25 °C using an OmniFluo-900 Steady-State and Time-Resolved Fluorescence Spectrometer (ZOLIX Instruments Co., Ltd., Beijing, China). The samples were excited by 355 nm light with a pulse width of 16 ps. Fluorescence emission at 460 nm was detected as a function of time via time-correlated single photon counting. The 355 nm incident light was obtained from an SSP-PLFL-335-50mW-3-20 laser (CNI Laser, Changchun New Industries Optoelectronics Tech. Co., China) operating at 20 MHz. The instrument response function was measured as scattered excitation light with the emission monochromator tuned to the excitation wavelength using the pure buffer. The fluorescence decay curves were analysed via iterative deconvolution in the OmniFluo software (version 1.2.28, ZOLIX Instruments Co., Ltd., China).

### 3.6. Data Analysis

Pre-steady-state kinetic data were subjected to numerical fitting using the DynaFit software 4.0 (BioKin, Pullman, WA, USA) [40,41] as described elsewhere [42,43].

Data from single-turnover PAGE experiments were fitted to a single-exponent equation that measures the rate of dNTP incorporation (*k*^obs^) per given dNTP concentration ([dNTP]). These data can then be utilised to determine *K*_d_ (the dissociation constant for the binding of dNTP to the enzyme–primer binary complex) and *k*^pol^ (the maximum rate of chemical catalysis). This was done by fitting the data to Equation (2). Kinetic parameters were obtained by numerical fitting in the OriginPro 7.5 software (OriginLab, Northampton, MA, USA).

*k*^obs^ = (*K*^dNTP^_d_ × *k*pol × [dNTP]_0_)/(*K*^dNTP^_d_ × [dNTP]_0_ + 1) (2)

### 3.7. Protein Sequence Alignment

Sequences of all InterPro IPR027292 TdT family members (469 enzymes) were retrieved from UniProt and aligned using MAFFT G-INS-I [44,45,46]. The resulting alignment was stripped of duplicates, fragments and isoforms. Individual TdT orthologs were manually picked to present the discussed amino acid positions. Select PolX homologs were aligned in MAFFT E-INS-i. Alignment images were generated in Jalview [47].

## 4. Conclusions

The conformational dynamics of TdT plays a major role in the incorporation of dNTP during the catalytic cycle. The interaction of pre-formed complex [E•DNA_ss_] with dNTP involves a two-step binding of dNTP followed by the catalytic step. It should be pointed out that the nature of the nitrogenous base affects both the binding process and the catalytic step of the enzymatic process. For instance, purine nucleoside triphosphates form an initial complex with higher *K*_1_ as compared to pyrimidine nucleoside triphosphates. The second equilibrium stage seems to match a conformational adjustment of the enzyme to the dNTP being incorporated, the formation of specific contacts with its nitrogenous base and the assembly of a catalytically active complex. This stage is most efficient for ddCTP and ddGTP, whereas for ddATP, *K*_2_ is the lowest. The catalytic rate constant is the highest for ddGTP; this property, along with the efficient course of the first two steps, makes dGTP the best substrate for TdT.

Our analysis of the change in C^Py^ fluorescence intensity during the interaction of dC^Py^TP with WT TdT in the absence of a DNA primer also points to the presence of two equilibrium states. Meanwhile, the second stage is characterised by a high association constant *K_2_*, indicating the stability of the final [E•dC^Py^TP]_2_ complex. In the presence of a DNA primer, the formation of the second complex proceeds faster (*k*_2_ for the binding of dC^Py^TP is 1.6 and 4.2 s^−1^, respectively).

The processivity of primer extension (based on the following: after the catalytic step, quick binding and rapid attachment of the next nucleoside triphosphate take place) is determined by the network of contacts between the incoming dNTP and amino acid residues in the active-site pocket. It is the binding of dNTP and its correct positioning in the active site that are key to the formation of the catalytically active complex, and these steps considerably influence both the efficiency of attachment of nucleotides of different structures and the processivity of this attachment. Furthermore, in the presence of surrogate ions Co^2+^ or Mn^2+^, switching to the processive mode of primer elongation occurs, regardless of the nature of the nucleoside triphosphate.

Analysis of the architecture of the dNTP-binding pocket and examination of amino acid sequence alignments for nucleotidyl transferases from 469 species of organisms revealed several potentially important active-site amino acid residues, among which residues Asp395 and Glu456 can determine the specificity of the enzyme for the nucleotide being incorporated. To determine the functional role of these residues in implementation of substrate specificity, mutants of TdT containing a single substitution (D395N, D395E or E456N) were assayed, as were double mutants of TdT (containing D395N/E456N or D395K/E456Q substitutions). Substitutions of Asp395 leading to neutralisation, relocation or inversion of the charge resulted in slightly lower activity of the enzyme. By contrast, charge neutralisation and an increase in the internal volume of the active site by E456N or D395N/E456N substitutions induced a transition to the processive mode and caused a substantial increase in the activity of the enzyme in the presence of Mg^2+^ ions.

Thus, our data show complicated multiparametric control of TdT activity, and that this control depends both (i) on the steps of dNTP binding in the active site and (ii) on the rate of catalytic attachment of the incoming nucleotide to the growing strand. In turn, both processes are affected by the nature of the incoming nucleotide (which induces successive conformational rearrangements in the enzyme) and the overall phenomenon of charge neutralisation and expansion of the internal volume in the dNTP-binding pocket, and, finally, by the nature of the cofactor metal ions, which all together regulate the switching between the distributive and processive mode of primer elongation. Therefore, it can be theorised that the high flexibility of the enzyme’s efficiency is a feature ‘programmed’ by nature, and that the combination of these factors ensures efficient execution of the biological function of this enzyme during the filling of DNA ends in V(D)J recombination.

## Figures and Tables

**Table 1:** Rate constants for interactions of the [WT TdT•DNA] complex with ddNTP.

Constants	ddGTP	ddTTP	ddATP	ddCTP
*k*_1_, (µM·s)^−1^	19 ± 1.8	7.2 ± 2	20 ± 0.8	16 ± 3
*k*_−1_, s^−1^	15 ± 1.0	18 ± 1.8	2.1 ± 0.05	40 ± 4
*K*_1_, µM^−1^	1.3 ± 0.2	0.4 ± 0.15	9.2 ± 0.6	0.4 ± 0.1
*k*_2_, s^−1^	22 ± 2.0	5.6 ± 1.8	0.17 ± 0.07	19 ± 1.5
*k*_−2_, s^−1^	20 ± 2.0	12 ± 0.9	3.0 ± 0.1	9.5 ± 0.8
*K*_2_	1.1 ± 0.2	0.45 ± 0.2	0.06 ± 0.01	2 ± 0.3
*K*^dNTP^_d_, µM	0.4 ± 0.1	1.7 ± 0.8	0.1 ± 0.03 ^Trp^ 0.37 ± 0.13 ^PAAG^	0.8 ± 0.2
*k*_pol_, s^−1^	2.4 ± 0.4	0.5 ± 0.1	0.1 ± 0.03 ^PAAG^	1.2 ± 0.2

*K*^dNTP^_d_ = 1/(*K*_1_ + *K*_1_ × *K*_2_), *K*_i_ = *k*_i_/*k*_−i_, *K*^dATP, PAAG^_d_ and *k*^dATP, PAAG^_pol_ were determined via Equation (2). PAAG: polyacrylamide gel.

**Table 2:** Rate constants for interactions of TdT or of the [TdT•DNA] complex with dC^Py^TP.

Constants	dC^Py^TP Binding	dC^Py^TP Incorporation	
		Fluorescence Analysis	PAGE Analysis
*k*_1_, (µM·s)^−1^	2.9 ± 0.5	3.6 ± 0.4	NA
*k*_−1_, s^−1^	11.0 ± 0.4	4.5 ± 0.3	NA
*K*_1_, µM^−1^	0.26 ± 0.06	0.8 ± 0.1	NA
*k*_2_, s^−1^	1.6 ± 0.15	4.2 ± 0.2	NA
*k*_−2_, s^−1^	0.15 ± 0.02	2.5 ± 0.1	NA
*K*_2_	11.0 ± 2.4	1.7 ± 0.1	NA
*K*^dCPyTP^_d_, µM	0.3 ± 0.1	0.5 ± 0.1	1.8 ± 0.7
*k*_pol_, s^−1^	NA	3.3 ± 0.2	3.6 ± 0.5

*K*^dCPyTP^_d_ = 1/(*K*_1_ + *K*_1_ × *K*_2_); *K*_i_ = *k*_i_/*k*_−i_; NA: not applicable.

**Table 3:** Occurrence of amino acid residues Asp395, Leu397, Phe404, Trp449, Arg453, Glu456 and Arg457 among TdT enzymes from 469 species.

Amino Acid Residue of Human TdT	Frequency Profile of Amino Acid Residues, %
Asp395	Asp 72.3%, Glu 27.3%, Asn 0.2% (1 of 469), Lys 0.2% (1 of 469)
Leu397	Leu 45.2%, Met 50.7%, Phe 4.1%
Phe404	Phe 100%
Trp449	Trp 100%
Arg453	Arg 91.4%, Thr 6.3%, Lys 1% (5 of 469), Pro 1% (5 of 469)
Glu456	Glu 76.3%, Gly 21.1%, Asp 1.5% (7 of 469), Leu 0.6% (3 of 469)
Arg457	Arg 99.8%, Lys 0.2% (1 of 469)

## Data Availability

Experimental data are available upon request to N.A.K. Tel.: +7-(383)-363-5175, E-mail: nikita.kuznetsov@niboch.nsc.ru.

## Supplementary Materials

The following supporting information can be downloaded at: https://www.mdpi.com/article/10.3390/ijms25020879/s1.
