# Supplementary material for: Substrate Specificity Diversity of Human Terminal Deoxynucleotidyltransferase May Be a Naturally Programmed Feature Facilitating Its Biological Function
Source: Int J Mol Sci. 2024 Jan 10;25(2):879. doi: 10.3390/ijms25020879 (PMC10815903; doi:10.3390/ijms25020879)
Supplement: Supplementary file 1 [file ijms-25-00879-s001.zip › ijms-2775247-supplementary/SUPPLEMENTARY DATA.pdf]

SUPPLEMENTARY DATA

Substrate specificity diversity of human terminal deoxynucleotidyltransferase may be a naturally programmed feature facilitating its biological function

Aleksandra A. Kuznetsova, Svetlana I. Senchurova, Anastasia A. Gavrilova, Timofey E. Tyugashev, Elena S. Mikushina, Nikita A. Kuznetsov

Institute of Chemical Biology and Fundamental Medicine, Siberian Branch of Russian Academy of Sciences (SB RAS), 8 Prospekt Akad. Lavrentyeva, Novosibirsk 630090, Russia

*To whom correspondence should be addressed: N.A.K. Tel.: +7 (383) 363-5175, Email: nikita.kuznetsov@niboch.nsc.ru

Table S1. The positions of amino acid substitution and the primer sequences for mutation.

Amino acid substitution	Primer	Sequences
D395E	forward	5'-GTTGCCTAGCAGGAAGGTTGAAGCTTTGGATCATTTTC-3'
	reverse	5'-GAAAATGATCCAAAGCTTCAACCTTCCTGCTAGGCAAC-3'
D395K	forward	5'-CAGGTTGCCTAGCAGGAAGGTTAAAGCTTTGGATCATTTTC-3'
	reverse	5'-GAAAATGATCCAAAGCTTTAACCTTCCTGCTAGGCAACCTG-3'
D395N	forward	5'-GTTGCCTAGCAGGAAGGTTAATGCTTTGGATCATTTTC-3'
	reverse	5'-GAAAATGATCCAAAGCATTAACCTTCCTGCTAGGCAAC-3'
E456Q	forward	5'-CTGGCTCCCGGCAGTTTCAGAGAGACCTCCGGCGCTATG-3'
	reverse	5'-CATAGCGCCGGAGGTCTCTCTGAAACTGCCGGGAGCCAG-3'
E456N	forward	5'-CTGGCTCCCGGCAGTTTAATAGAGACCTCCGGCGCTATG-3'
	reverse	5'-CATAGCGCCGGAGGTCTCTATTAAACTGCCGGGAGCCAG-3'
